# Detection of serum interleukin-18 level and neutrophil/lymphocyte ratio in patients with antineutrophil cytoplasmic antibody-associated vasculitis and its clinical significance

**DOI:** 10.1515/biol-2022-0823

**Published:** 2024-02-05

**Authors:** Changning Liu

**Affiliations:** Department of Laboratory Medicine, Key Laboratory of Precision Medicine for Viral Diseases, Guangxi Health Commission Key Laboratory of Clinical Biotechnology, Liuzhou People’s Hospital, Liu Zhou 545006, China

**Keywords:** antibody-associated vasculitis, interleukin-18, autoimmune diseases, immunological response, inflammatory homeostasis

## Abstract

Anti-neutrophil cytoplasmic antibody (ANCA)-associated vasculitis (AAV) is a group of autoimmune diseases. This study aimed to investigate the clinical significance of changes in interleukin-18 (IL-18) and neutrophil/lymphocyte ratio (NLR) in the pathogenesis of AAV and the impact of NLR on the prognosis of patients. The clinical data of 52 AAV patients (AAV group) who met the conditions of hospitalization, 30 patients with mild mesangial proliferative glomerulonephritis (disease controls), and 30 healthy volunteers (normal controls) in Nephrology Department of Liuzhou People’s Hospital from May 2020 to August 2022 were selected. A total of 52 AAV patients were divided into active phase (>15 points) and remission phase (≤15 points) based on the Birmingham vasculitis activity score (BVAS). Serum IL-18 level was detected by enzyme-linked immunosorbent assay in three groups. Pearson product moment correlation analysis was performed to investigate the correlation between serum IL-18 levels and clinical laboratory indicators, and receiver operating characteristic (ROC) curve analysis was performed on serum IL-18, C-reactive protein (CRP), erythrocyte sedimentation rate (ESR) levels, and NLR in AAV patients. The levels of serum creatinine, parathyroid hormone, β2-microglobulin (β2-MG), ESR, CRP, and IL-18 in active stage of AAV were significantly higher than those in remission stage of AAV. Moreover, the serum IL-18 level of active AAV patients was significantly higher than that of disease control group (*P* < 0.05). The levels of eGFR, hemoglobin, and complement C3 were significantly lower than those during the remission (*P* < 0.05). Pearson product moment correlation analysis showed that serum IL-18 level in AAV patients was positively correlated with BVAS score and ESR level. The area under the curve of serum IL-18, NLR, CRP, ESR levels evaluated by ROC curve was 0.921, 0.899, 0.83, and 0.75, respectively. Kaplan–Meier survival curve showed that the cumulative survival rate of patients in low NLR group was significantly higher than that in high NLR group (68.36 vs 42.89%), with significant difference (Log-Rank = 6.745, *P* = 0.025 < 0.05). IL-18 may be adopted as one of the important biological markers to judge the disease of AAV, and the cumulative survival rate of patients with high NLR is low, which may be applied as an indicator to evaluate the poor prognosis of patients with AAV.

## 1 Introduction

Anti-neutrophil cytoplasmic antibody (ANCA)-associated vasculitis (AAV) is a group of autoimmune systemic diseases, mainly characterized by fibrinoid inflammation of small vessels as the main pathological feature, which is caused by various factors such as genetics and environment, endothelial cells, ANCA and target antigens, and neutrophils [1–3]. AAV often involves the kidney, i.e., ANCA-associated glomerulonephritis (AGN), and is one of the common critical illnesses in clinical practice [4–7].

Interleukin-18 (IL-18) is a new type of Th1 cytokine, which can induce helper T cells and natural killer cells to produce interferon-γ (IFN-γ) and was first defined as an IFN-γ inducing factor [8–11]. IL-18 expression is upregulated in many autoimmune diseases, for example, autoimmune hepatitis, rheumatoid arthritis, type I diabetes, multiple sclerosis, and other diseases [12–16]. In recent years, the literature suggests that IL-18 can be used as one of the serum markers of acute kidney injury, and its expression is increased in the renal tissue of AAV patients [17].

ANCA mainly includes proteinase-3 antibody (PR3-ANCA), myeloperoxidase antibody (MPO-ANCA), and lysosomal-associated membrane protein-2 antibody (LAMP-2), which are important serological markers for assessing AAV activity [18–21]. In addition, immune cells such as neutrophils and lymphocytes are involved in the pathogenesis of AAV. Neutrophil lymphocyte ratio (NLR) combines immune pathways of two different factors, and the larger the calculated value means that the body’s inflammatory homeostasis is more likely to be compromised [22–26]. NLR is associated with cancer, cardiovascular disease, diabetes, and hypertension, and can be used as a systemic inflammatory marker to independently predict the prognosis of cancer, coronary heart disease, and other diseases.

IL-18 plays an important role in the immune response, especially in promoting inflammation and regulating the immune response, produced by various immune cells such as monocytes, macrophages, and dendritic cells. Studies have shown that in ANCA-associated vasculitis, the level of IL-18 may increase, which may be due to the activation of immune cells during the inflammatory process leading to the release of IL-18 [27]. The NLR is usually used to reflect the inflammatory state of the body, while ANCA-associated vasculitis is usually accompanied by a significant inflammatory response. The increase in NLR may reflect an increase in neutrophils, and the body is undergoing an inflammatory process [28,29]. IL-18 and NLR are both biomarkers associated with inflammation. In inflammatory diseases such as ANCA-associated vasculitis, the inflammatory response is usually very active, so these two markers may be helpful in evaluating the degree of disease activity. Therefore, the clinical data of 52 patients with AAV were retrospectively analyzed. Receiver operating characteristic (ROC) curve analysis was carried out for serum IL-18, C-reactive protein (CRP), and erythrocyte sedimentation rate (ESR) levels in patients with AAV to investigate the clinical significance of serum IL-18 levels in pathogenesis of AAV and the effect of NLR changes on the prognosis.

## 2 Subjects and methods

### 2.1 Study subjects

The clinical data of 52 patients with AAV in Nephrology Department of Liuzhou People’s Hospital from May 2020 to August 2022 were selected as the AAV group and a retrospective study was performed to diagnose renal injury, 28 men and 24 women, and the mean age was (54.8 + 13.7). According to Birmingham vasculitis activity score (BVAS), patients were divided into AAV AS (27 patients) and AAV RS (25 patients). Patients were divided into two groups for analyzing the prognosis effect of NLR: high NLR group (NLR ≥ 3.77) and low NLR group (NLR < 3.77) using the median NLR of 3.77 as the cut-off point.

Thirty inpatients during the same period were selected as the disease controls, mainly manifested as proteinuria and microscopic hematuria, and renal histopathological biopsy confirmed the diagnosis of mild mesangial proliferative glomerulonephritis, composed of 12 men and 18 women, and the average age was (56.1 ± 4.7).

Thirty healthy volunteers without underlying diseases and with normal renal function from the Health Examination Center of Liuzhou People’s Hospital during the same period were selected as the healthy controls having 14 men and 16 women, mean age (51.3 + 11.4).

Patients with AAV were included and excluded as follows.

**Inclusion criteria:** (1) AAV patients according to the 2012 American Chapel Hill classification criteria for systemic vasculitis; (2) AAV positive, pauci-immune crescentic glomerulonephritis, and thick granular IgA deposition in mesangial area; and (3) clinical data and laboratory data were complete.

**Exclusion criteria:** (1) people with heart disease; (2) HIV infection or AIDS patients, pregnant, or lactating women (except for bacteria and fungi); (3) people with cancer, hepatitis; (4) people with other autoimmune diseases and primary kidney disease such as membranous nephropathy; and (5) people with drug-induced vasculitis, allergic purpura.


**Informed consent:** Informed consent has been obtained from all individuals included in this study.
**Ethical approval:** The research related to human use has been complied with all the relevant national regulations, institutional policies and in accordance with the tenets of the Helsinki Declaration, and has been approved by the authors’ institutional review board or equivalent committee.

### 2.2 Outcome measures

Clinical data were collected: age, gender, disease duration, body mass index (BMI, kg/m^2^), blood pressure, and pulse of the patients.

Laboratory data were collected: red blood cell distribution width (RDW), white blood cell count (WBC), platelet count (PC), Hb, absolute monocyte count (AMC), absolute neutrophil count (ANC), absolute lymphocyte count (ALC), Scr, blood uric acid, plasma albumin, ANCA, antinuclear antibody profile, liver and kidney function, CRP, β2-MG, ESR, complement C3, and 24 h urinary protein quantification (24hUP), all of which were detected by the clinical laboratory of the hospital. Granulocyte mosaic indirect immunofluorescence assay kit (Beijing Sanoulikang Biotechnology Co., Ltd) detected serum ANCA and anti-GBM antibodies. ANCA was detected adopting anti-MPO, PR3, and anti-glomerular basement membrane antibody IgG detection kits (Hunan Leidu Biotechnology Co., Ltd).

eGFR is computed as follows:

(1) \[\text{eGFR}=186\times (\text{SCr})-1.154\times \text{age}-0.203\times 0.742\hspace{.5em}(\text{female}),]\]

where SCr is in µmol/L, age in years, and eGFR in mL min^−1^ (1.73 m^2^)^−1^.

BVAS: If the total score is more than 15 points, it is AS; otherwise, it is RS.

### 2.3 Detecting IL-18 concentrations

Serum IL-18 levels were measured using a human IL-18 enzyme-linked immunosorbent assay (ELISA) kit (Beijing Solarbio Technology Co., Ltd), and the experimental operation was performed in strict accordance with the kit instructions to determine the absorbance (*A* value) of each well at a wavelength of 450 nm. The *A* value was adopted to make a standard curve by Curve expert software (provider: Beijing Boleide Technology Development Co., Ltd) to calculate the concentration of the corresponding samples.

### 2.4 Treatment

Initial treatment: oral prednisone 1 mg/(kg.day) for 4–6 weeks, reduced to 12.5–15 mg after 3 months, to 10 mg after 6–9 months, to 5 mg after 18–24 months, and even discontinued.

Methylprednisolone pulse therapy: 0.5–1 g once a day, 3 times/course for patients with acute kidney injury such as fibrinoid necrosis of small vessels, cellular crescent formation, or pulmonary hemorrhage, followed by oral prednisone.

Cyclophosphamide treatment regimen: oral 2 mg/(kg day), twice, for 2–4 months, or intravenous shock 0.5–0.7 g/m^2^, once/month for 7–9 months.

### 2.5 Follow-up

Observed primary composite end point events included end-stage renal disease (ESRD), doubling of Scr, and death adopting outpatient review questioning, telephone calls, and text messaging counseling as follow-up modalities.

### 2.6 Statistical methods

Data analysis was carried out applying SPSS 19.0 software. The scores that conformed to normal distribution were presented as ( \[\bar{x}]\] ± s), with *t* test, frequency and percentage (%) for clinical data, and *χ* ^2^ test used. Pearson product moment correlation was adopted, the area under the curve (AUC) of ROC to evaluate the accuracy of IL-18 as an indicator to assess AAV disease activity. Survival analysis was performed applying Kaplan–Meier (KM) method, which aimed to calculate SR. Log-Rank method was applied to compare SR between both groups. Statistical results of *P <* 0.05 were considered statistically significant.

## 3 Results

### 3.1 Clinical data

Twenty-seven patients had BVAS scores greater than 15, which were in AS; 25 patients had BVAS scores less than 15, which were in RS, 28 men and 24 women, mean age (54.8 + 13.7). All AAV patients presented with renal involvement, 25 patients had pulmonary involvement, 14 patients had fatigue, 5 had weight loss, 5 had fever, 3 had arthralgia, and 1 had myalgia. There was no statistical difference in gender, age, BMI (kg/m^2^), pulse (beats/min), systolic blood pressure (SBP) (mmHg), diastolic blood pressure (DBP) (mmHg), ANCA (MPO/PR3), disease duration, and organ involvement between both groups (*P >* 0.05) (Table 1).

**Table 1:** Statistics of clinical data of patients with AVV ( \[\bar{x}]\] ± s, *n* [%])

Item	AAV AS (*n* = 27)	AAV RS (*n* = 25)	*P*
**Gender**			
Male	17 (62.9)	11 (44.0)	>0.05
Female	10 (37.0)	14 (56.0)	
Mean age (years)	53.5 ± 12.7	54.3 ± 10.2	>0.05
BMI (kg/m^2^)	22.78 ± 2.76	21.84 ± 1.62	>0.05
Pulse (beats/min)	94.63 ± 17.35	89.26 ± 15.93	>0.05
SBP (mmHg)	145.26 ± 23.64	138.24 ± 18.35	>0.05
DBP (mmHg)	84.37 ± 14.26	78.36 ± 8.32	>0.05
ANCA (MPO/PR3)	25/2	24/1	>0.05
Disease duration (months)	5.73 ± 1.24	4.32 ± 1.02	>0.05
Renal involvement	27 (100)	25 (100)	>0.05
Pulmonary involvement	16 (59.2)	7 (28.0)	>0.05
Asthenia	12 (44.4)	2 (8.0)	>0.05
Decreased weight	2 (7.4)	3 (12.0)	>0.05
Fever	2 (7.4)	3 (12.0)	>0.05
Arthralgia	3 (11.1)	0 (0)	>0.05
Muscular pain	0 (0)	1 (4.0)	>0.05

Note: AS versus RS of AAV, *P <* 0.05.

### 3.2 Laboratory test results

Scr (578.43 ± 212.74 μmol/L vs 231.23 ± 89.21 μmol/L), parathyroid hormone (324.27 pg/mL vs 38.29 pg/mL), and β2-MG levels (11.25 ± 5.78 mg/L vs 6.83 ± 3.47 mg/L) were higher in AS of AAV; ESR (84.36 ± 27.35 mm/h vs 38.64 ± 24.14 mm/h) and CRP levels (35.2 mg/L vs 8.73 mg/L) were lower, eGFR values (12.31 mL/min/1.73 m^2^ vs 27.35 mL/min/1.73 m^2^) were higher in RS of AAV (*P <* 0.05). Hb (74.62 ± 16.26 g/L vs 98.35 ± 25.34 g/L) and complement C3 levels (0.68 ± 0.25 g/L vs 0.85 ± 0.21 g/L) were superior in patients with RS (*P <* 0.05) (Table 2).

**Table 2:** Laboratory test results of AVV ( \[\bar{x}]\] ± s)

Item	AAV AS (*n* = 27)	AAV RS (*n* = 25)	*P*
Scr (μmol/L)	578.43 ± 212.74	231.23 ± 89.21	<0.05*
Urea nitrogen (μmol/L)	18.26 ± 6.35	14.25 ± 12.63	
Blood uric acid (μmol/L)	427.31 ± 112.13	376.32 ± 121.26	
eGFR (mL/min/1.73 m^2^)	12.31	27.35	<0.05*
24hUP (g/24 h)	1.26 ± 1.24	1.21 ± 0.78	>0.05
ESR (mm/h)	84.36 ± 27.35	38.64 ± 24.14	<0.05*
CRP (mg/L)	35.2	8.73	<0.05*
Plasma albumin (g/L)	32.61 ± 4.26	35.62 ± 4.72	>0.05
WBC count (10^9^/L)	8.35 ± 2.15	8.21 ± 3.27	>0.05
PC (10^9^/L)	203.26 ± 83.75	256.37 ± 158.25	>0.05
Hb (g/L)	74.62 ± 16.26	98.35 ± 25.34	<0.05*
ANC (10^9^/L)	5.34 ± 2.48	5.26 ± 2.16	>0.05
ALC (10^9^/L)	1.25 ± 0.36	1.45 ± 0.25	>0.05
AMC (10^9^/L)	0.53 ± 0.21	0.48 ± 0.16	>0.05
Red cell distribution width (%)	14.26 ± 2.51	13.37 ± 1.48	>0.05
Complement C3 (g/L)	0.68 ± 0.25	0.85 ± 0.21	<0.05*
Parathyroid hormone (pg/mL)	324.27	38.29	<0.05*
β2-MG (mg/L)	11.25 ± 5.78	6.83 ± 3.47	<0.05*

Note: AS versus RS of AAV, *P < 0.05.

This was not clearly different in WBC count, urea nitrogen, 24hUP, plasma albumin, blood uric acid, PC, ANC, ALC, RDW, and AMC between both groups (*P >* 0.05).

### 3.3 Serum IL-18 levels and comparison

**Figure 1:**
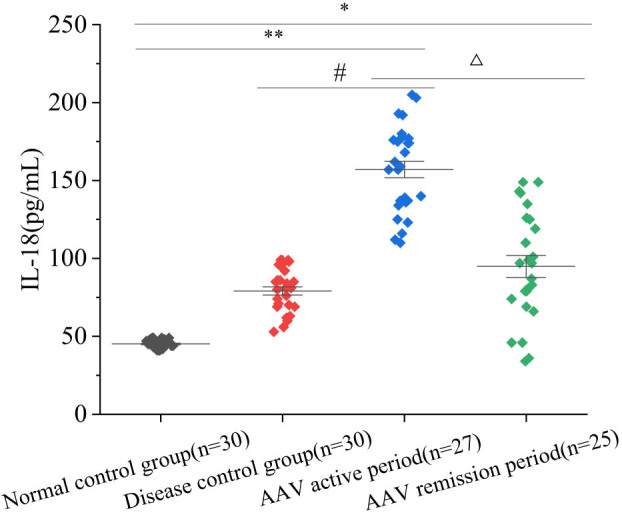
Comparison of serum IL-18 levels. Note: Compared with normal controls, *P < 0.05; relative to disease controls, ^#^ *P <* 0.05; comparison between AAV in AS and RS, ^△^ *P <* 0.05.

The experimental procedures were performed according to the instructions of the kit, and the OD values of the standards and samples were measured at 450 nm with a microplate reader, and the standard curve of serum IL-18 levels was drawn through Curve expert software to calculate the concentration in corresponding samples. The results suggested that the serum IL-18 level in active AAV patients was higher than that in AAV patients in RS and disease controls; they were higher in AAV patients in active and in RS than in healthy controls (*P <* 0.05). This was similar between disease controls and healthy controls (*P >* 0.05) (Figure 1).

### 3.4 Correlation analysis between serum IL-18 levels and clinical parameters

**Figure 2:**
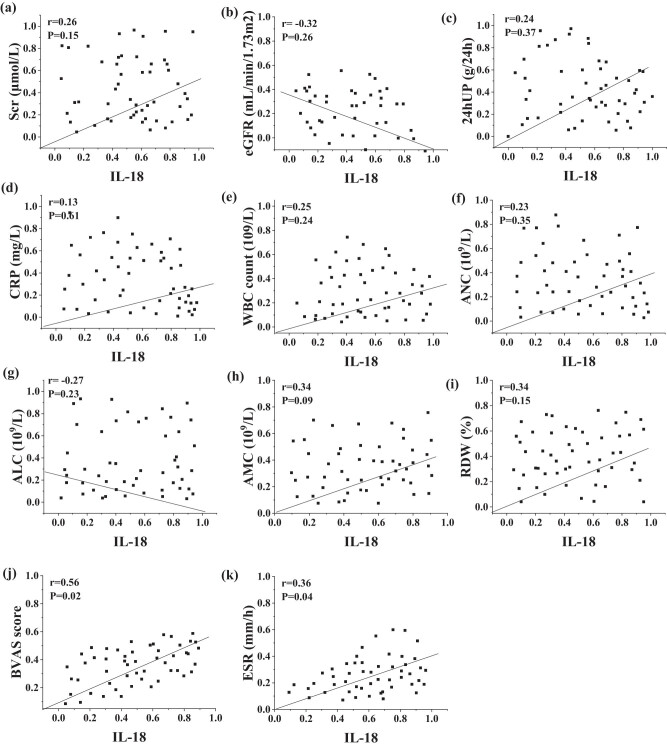
Correlation analysis of serum IL-18 levels with clinical indices (a–k represent the following indices in sequence: Scr, Egfr, 24hUP, CRP, WBC count, ANC, ALC, AMC, RDW, BVAS score, and ESR).

Pearson correlation analysis of the correlation indicated a positive relation between serum IL-18 levels and BVAS scores (*r* = 0.56, *P =* 0.02) and ESR levels (*r* = 0.36, *P =* 0.04) in AAV patients. IL-18 levels had no obvious correlation with Scr (*r* = 0.26, *P =* 0.15), eGFR (*r* = −0.32, *P =* 0.26), 24hUP (*r* = 0.24, *P =* 0.37), CRP (*r* = 0.13, *P =* 0.61), WBC count (*r* = 0.25, *P =* 0.24), ANC (*r* = 0.23, *P =* 0.35), ALC (*r* = −0.27, *P =* 0.02), AMC (*r* = 0.34, *P =* 0.09), and RDW (*r* = 0.34, *P =* 0.15) (Figure 2 and Table 3).

**Table 3:** Correlation analysis between serum IL-18 levels and clinical parameters

Indicators	Statistical value	Scr (μmol/L)	eGFR (mL/min/1.73 m^2^)	24hUP (g/24 h)	CRP (mg/L)	WBC count (10^9^/L)	ANC (10^9^/L)
	*r*	0.26	−0.32	0.24	0.13	0.25	0.23
	*P*	0.15	0.26	0.37	0.61	0.24	0.35
Indicators	Statistical value	ALC (10^9^/L)	AMC (10^9^/L)	RDW (%)	BVAS score	ESR (mm/h)	
	*r*	−0.27	0.34	0.34	0.56	0.36	
	*P*	0.23	0.09	0.15	0.02	0.04	

### 3.5 ROC analysis of serum IL-18, CRP, and ESR levels to assess activity in AAV patients

**Figure 3:**
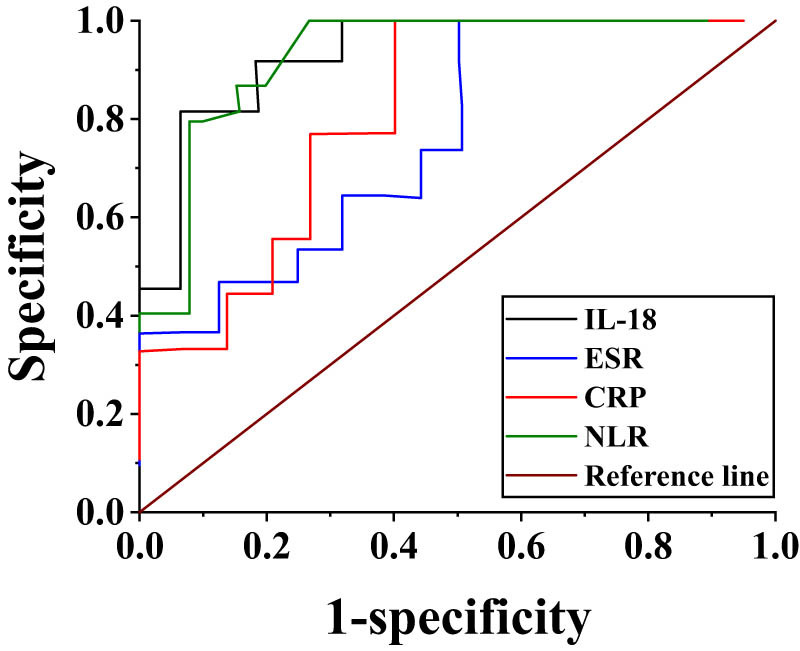
ROC analysis of serum IL-18, CRP, and ESR levels to assess activity in AAV patients.

ROC curve analysis indicated that the AUC of serum IL-18, serum CRP, and ESR levels for assessing activity in AAV patients was 0.921, *P <* 0.05, 95% CI (0.8323–1.0253; 0.83, *P <* 0.05, 95% CI (0.6457–0.9873); and 0.75, *P <* 0.05, 95% CI (0.5628–0.9671), respectively. The AUC for assessing the activity of AAV patients at NLR level was 0.899, *P* < 0.05, 95% CI (0.8562–0.9998). The AUC of IL-18 for assessing activity in AAV patients was greater than that corresponding to CRP and ESR and NLR, indicating that the accuracy of serum IL-18 levels assessment of activity in AAV patients was better. When the IL-18 level of 136.42 pg/mL was used as the cutoff value to assess the activity of AAV patients, its Sen and Spe were 91.26 and 86.7% (Figure 3).

### 3.6 Difference of NLR values between AAV patients and healthy controls

**Figure 4:**
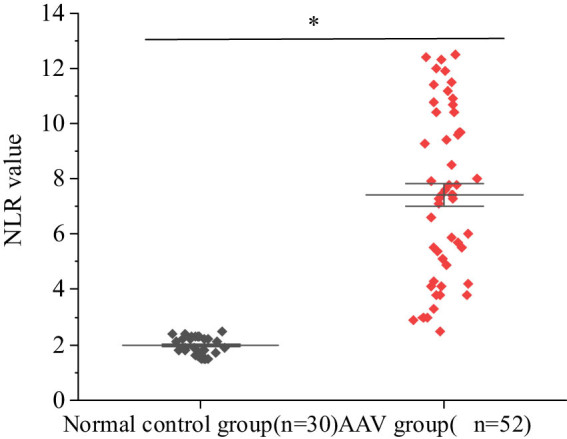
Comparison of NLR values between AAV patients and healthy controls.

Patients in AAV group had higher NLR values than healthy controls (*P <* 0.05) (Figure 4).

### 3.7 Comparison of laboratory tests in patients in different groups

In contrast with the low NLR group, WBC count (median 5.37 × 10^9^/L vs 8.75 × 10^9^/L, *P <* 0.05), AMC (0.31 × 10^9^/L vs 0.46 × 10^9^/L), ANC (0.46 × 10^9^/L vs 6.56 × 10^9^/L), PLR (141.50 vs 267.42), CRP level (4.35 g/L vs 41.15 g/L), and BVAS score (12.43 ± 2.36 vs 17.52 ± 4.59) were increased in high NLR group (*P <* 0.05).

The median value of eGFR was decreased in high NLR group relative to the low NLR group (76.19 mL/min/1.73 m^2^ vs 14.87 mL/min/1.73 m^2^, *P <* 0.05).

This was similar in RDW, ALC, Scr, Hb, PC, blood uric acid, ESR, plasma albumin, complement C3, 24hUP, and ANCA distribution between both groups (*P >* 0.05) (Table 4).

**Table 4:** Comparison of laboratory tests in patients in different groups

Item	Low NLR group (*n* = 27)	High NLR group (*n* = 25)	*P*
WBC count (10^9^/L)	5.37 (4.56–6.84)	8.75 (6.45–11.26)	<0.05*
Red blood cell width (%)	13.46 (12.5–14.2)	13.78 (12.53–14.35)	>0.05
Hb (g/L)	104.36 ± 21.53	84.76 ± 18.65	>0.05
PC (10^9^/L)	236.00 (184.00–267.00)	236.00 (169.00–295.00)	>0.05
Absolute monocytes count (10^9^/L)	0.31 (0.24–0.48)	0.46 (0.33–0.61)	<0.05*
Absolute neutrophils count (10^9^/L)	0.46 (0.35–0.56)	6.56 (5.35–9.89)	<0.05*
ALC (10^9^/L)	1.65 ± 0.41	0.98 ± 0.42	>0.05
PLR	141.50 (136.95–172.64)	267.42 (157.59–334.53)	<0.05*
eGFR (mL/min/1.73 m^2^)	76.19 (11.14–123.38)	14.87 (11.13–27.66)	>0.05
Scr (μmol/L)	138.10 (74.70–542.00)	378.70 (256.80–591.00)	>0.05
Blood uric acid (μmol/L)	367.39 ± 113.97	467.87 ± 121.55	>0.05
Plasma albumin (g/L)	36.38 ± 7.59	32.79 ± 4.63	>0.05
24hUP (g/24 h)	1.56 (0.51–3.47)	1.02 (0.51–2.83)	>0.05
ESR (mm/h)	67.31 ± 25.48	83.32 ± 33.44	>0.05
CRP (g/L)	4.35 (3.28–8.57)	41.15 (14.47–98.53)	<0.05*
Complement C3 (g/L)	0.78 ± 0.23	0.76 ± 0.32	>0.05
MPO-ANCA/PR3-ANCA	24/3	21/4	>0.05
BVAS score	12.43 ± 2.36	17.52 ± 4.59	<0.05*

Note: Low NLR group versus high NLR group, *P < 0.05.

### 3.8 Comparison of treatment regimens for patients in different groups

Fewer patients in low NLR group received steroid pulse therapy (22.22 vs 64.00%) (*P* < 0.05). Other treatment regimens had no obvious distinction between both groups (*P* > 0.05) (Table 5).

**Table 5:** Comparison of treatment regimens for patients in different groups (*n* [%)])

Treatment regimen	Low NLR group (*n* = 27)	High NLR group (*n* = 25)	*P*
Hormone alone	8 (29.62)	11 (44.00)	0.736
Hormone + Cyclophosphamide	6 (22.22)	11 (44.00)	0.342
Hormone + Cyclophosphamide (IV)	3 (11.11)	6 (24.00)	0.425
Hormone + Cyclophosphamide (PO)	5 (18.51)	6 (24.00)	1.000
Hormone + tacrolimus	1 (3.70)	2 (8.00)	1.000
Hormone + mycophenolate mofetil	1 (3.70)	1 (4.00)	1.000
Hormone + triptolide	3 (11.11)	2 (8.00)	1.000
Hormone pulse	6 (22.22)	16 (64.00)	0.015*
Immediate dialysis	5 (18.51)	5 (20.00)	1.000

Note: IV: intravenous drip; PO: oral. AS versus RS of AAV, *P <* 0.05.

### 3.9 Prognostic analysis of AAV in patients

**Figure 5:**
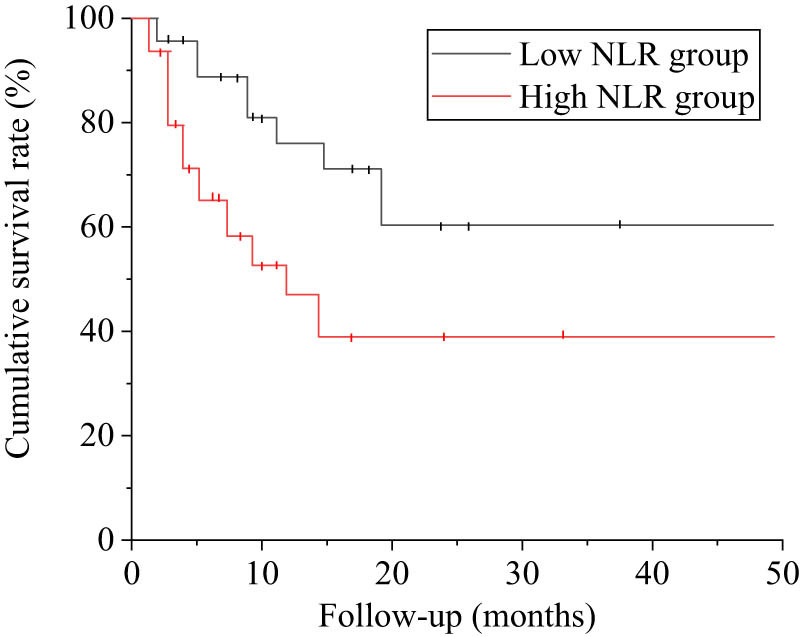
KM survival plots.

In low NLR group, the following visit time ranged from 1.7 to 48.5 (median 12.4 [4.8–21.5] months); in high NLR group, the following visit time ranged from 1.4 to 47.5 (4.6 [2.4–13.7] months). KM survival curves showed that patients in low NLR group had a higher cumulative survival rate (68.36 vs 42.89%) (Log-Rank = 6.745, *P* = 0.025 < 0.05) (Figure 5).

## 4 Discussion

AAV is an autoimmune disease, with features of fibrinoid necrosis of small vessels and crescentic shape, which involves multiple organs. As a serological marker, ANCA, although it can accurately diagnose AAV, it cannot reflect the activity of AAV [30–33]. Therefore, a novel marker needs to be sought to reflect the activity, recurrence, as well as prognosis of AAV. Serum IL-18 level in AAV patients was measured by ELISA, and its correlation with clinical data was analyzed. The results confirmed that IL-18 was correlated with AAV activity and could be used as a marker to assess AAV activity.

IL-18 promotes the generation of oxides in neutrophils bound to ANCA. Zhu et al. [34] found it was up-regulated in the affected renal tissue of AAV patients, mainly distributed in podocytes, and in addition, it was also slightly distributed in renal interstitial myofibroblasts, distal tubule epithelial cells, and infiltrating macrophages. Deshayes et al. [35] found that serum IL-18 levels were higher in patients with active granulomatous polyangiitis than in normal controls and patients in RS using electrochemiluminescence; Muto et al. [36] suggested that IL-18 is involved in AAV activity. In this experiment, the subjects included AAV group, disease controls, and healthy controls, and then AAV was further divided into AS and RS according to the BVAS score. It revealed that the serum IL-18 levels in AAV patients in AS and in RS were higher than those in healthy controls, and the levels in AAV patients in AS were higher than those in AAV patients in RS and disease controls (*P <* 0.05). This is consistent with the findings of Li et al. [37]. Pearson correlation analysis revealed a positive correlation between serum IL-18 levels and BVAS scores (*r* = 0.56, *P =* 0.02 < 0.05) and ESR levels (*r* = 0.36, *P =* 0.04 < 0.05) in AAV patients. Serum IL-18 levels had no obvious correlation with Scr, eGFR, 24hUP, WBC count, absolute neutrophil, absolute lymphocyte, absolute monocyte values, CRP, and RDW. Further ROC curve analysis suggested that the AUC of IL-18 for assessing activity in AAV patients was greater than that corresponding to CRP and ESR and NLR, meaning that the accuracy of serum IL-18 level for assessing activity in AAV patients was better. It indicated the level may be correlated with the activity of AAV disease and is expected to be one of the new biological markers for assessing AAV activity.

A variety of immune cells such as neutrophils, T lymphocytes, and B lymphocytes mediate the pathological process of AAV. In immune diseases, neutrophils and lymphocytes are activators and regulators of inflammation, respectively, and NLR is an immune function that combines the above two different factors and reflects the patient’s own inflammation more than the above single factor, and the higher this ratio, the higher the probability that the body homeostasis is disrupted [38,39]. The present results revealed that patients in AAV group had higher NLR values than healthy controls (*P <* 0.05), suggesting that AAV patients may be in a condition of persistent inflammation. NLR is commonly used to assess the prognosis of cardiovascular diseases and tumors, and Ahmad et al. [40] found that remission of clinical symptoms of systemic inflammatory response occurs simultaneously with decreased neutrophils and elevated lymphocytes, and if the maintenance time of neutrophil increase and lymphocyte decrease exceeds 7 days, the body will have serious complications. Additionally, the low NLR group had fewer patients receiving hormone shock therapy than the high NLR group (22.22 vs 64.00%), indicating that low NLR levels represented good patient recovery. Therefore, high NLR can be applied as one of the important indicators of poor prognosis. However, the follow-up time of this study may be limited, and further exploration of related studies may require a longer follow-up time.

Studies have reported that the overall prognosis of AAV is poor, and the 1-year mortality rate of patients with active AAV is about 85% if no effective treatment is performed. Mortality was not clearly distinct between low and high NLR groups, and one patient each died of pulmonary infection, which may be due to the short following visit time and the small sample size. KM survival curves showed the cumulative survival rate of patients in low NLR group was superior (68.36 vs 42.89%) (Log-Rank = 6.745, *P =* 0.025 < 0.05). These results suggest that high NLR may be adopted as an indicator for assessment of the poor prognosis.

## 5 Conclusion

IL-18 may be an important biological marker to assess the disease nature of AAV; patients with high NLR have a low cumulative survival rate, which may assess the poor prognosis of AAV patients as an indicator. However, there are some shortcomings in this study: first, the sample size is limited, so it is necessary to expand the sample size and conduct multicenter validation in the future. Second, the follow-up time is limited and there is a lack of comprehensive understanding of the disease progression of the study subjects. To further explore the correlation between NLR levels and the survival prognosis of AAV patients, longer term follow-up and in-depth analysis are needed.
